# The Synergistic Anti-Cancer Effects of NVP-BEZ235 and Regorafenib in Hepatocellular Carcinoma

**DOI:** 10.3390/molecules25102454

**Published:** 2020-05-25

**Authors:** Cheng-Chan Yu, Sung-Ying Huang, Shu-Fang Chang, Kuan-Fu Liao, Sheng-Chun Chiu

**Affiliations:** 1 Department of General Surgery, Taichung Tzu Chi Hospital, Buddhist Tzu Chi Medical Foundation, Taichung 427213, Taiwan; jefferyu@gmail.com; 2 Department of Ophthalmology, Hsinchu Mackay Memorial Hospital, Hsinchu 300044, Taiwan; hopes929@gmail.com; 3 Department of Research, Taichung Tzu Chi Hospital, Buddhist Tzu Chi Medical Foundation, Taichung 427213, Taiwan; fantac10@gmail.com; 4 Department of Internal Medicine, Taichung Tzu Chi Hospital, Buddhist Tzu Chi Medical Foundation, Taichung 427213, Taiwan; 5 Department of Laboratory Medicine, Taichung Tzu Chi Hospital, Buddhist Tzu Chi Medical Foundation, Taichung 427213, Taiwan; 6 General Education Center, Tzu Chi University of Science and Technology, Hualien 973302, Taiwan

**Keywords:** regorafenib, BEZ235, hepatocellular carcinoma, PI3K/Akt/mTOR pathway, metastasis, combination therapy

## Abstract

Hepatocellular carcinoma (HCC) is the most common type of liver cancer worldwide. Regorafenib is a multi-kinase inhibitor and the second-line treatment for HCC. Since the PI3K/Akt/mTOR signaling pathway is dysregulated in HCC, we evaluated the therapeutic effects of regorafenib combined with a dual PI3K/mTOR inhibitor BEZ235 in the human HCC cell lines (*n* = 3). The combined treatment with BEZ235 and regorafenib enhanced the inhibition of cell proliferation and increased the expression of cleaved caspase-3 and cleaved PARP in HCC cells. Moreover, the combined treatment suppressed HCC cell migration and invasion in the transwell assay. Further, the Western blot analyses confirmed the involvement of epithelial-mesenchymal transition (EMT)-related genes such as slug, vimentin, and matrix metalloproteinase (MMP)-9/-2. Additionally, the proteinase activity of MMP-9/-2 was analyzed using gelatin zymography. Furthermore, the inhibition of phosphorylation of the Akt, mTOR, p70S6K, and 4EBP1 after combined treatment was validated using Western blot analysis. Therefore, these results suggest that the combined treatment with BEZ235 and regorafenib benefits patients with HCC.

## 1. Introduction

Hepatocellular carcinoma (HCC) is the fifth most common cancer and the third leading cause of cancer-related deaths globally [1]. HCC is considered to be a complex malignancy caused by alternations in multiple signaling pathways that are involved in cell proliferation and angiogenesis [2]. Moreover, the Ras/Raf/MAPK pathway is activated as a result of stimulation by growth factors, such as EGF, HGF, and IGF that are involved in the pathogenesis of HCC [3]. Sorafenib, a multi-kinase inhibitor (MKI), inhibits the progression in HCC by blocking the Ras/Raf/MAPK pathway and has demonstrated a significant improvement in the clinical outcome of patients with advanced HCC [4]. Conversely, regorafenib, a novel oral MKI, blocks several protein kinases involved in the tumor angiogenesis (VEGFR-1, -2, and -3), tumor growth (KIT, RET, RAF-1, and BRAF), and metastasis (PDGFR-β, FGFR1) of cancer cells [5,6,7]. Moreover, regorafenib has improved the overall survival (OS) of the HCC patients and shows fewer side-effects than sorafenib. Thus, it has been FDA-approved for the treatment of HCC patients who had poorly responded to treatment with sorafenib [8,9]. However, drug toxicity, resistance, and relapse continue to be problems in the treatment of patients with HCC. Therefore, it is necessary to identify combined strategies to improve the therapeutic effect of regorafenib in HCC.

The phosphatidylinositol 3-kinase (PI3K)/protein kinase B (Akt)/mammalian target of rapamycin (mTOR) signaling pathway plays crucial roles in regulating the cellular processes, including cell proliferation, survival, differentiation, angiogenesis, and apoptosis that are dysregulated in the drug-resistant HCC cells [10,11,12]. For instance, the proteins downstream of the mTOR signaling, such as the 70-kD ribosomal protein S6 kinase 1 (p70S6K1), regulate cell proliferation, cell cycle progression, and cell migration, and were found to be deregulated in 50% of the HCC cases [13,14,15]. Thus, targeting the PI3K/Akt/mTOR signaling pathway may serve as a novel therapy in HCC. The NVP-BEZ235 (BEZ235, also known as dactolisib), an imidazo[4,5-c]quinoline derivative, interacts with the ATP-binding cleft of enzymes to suppress PI3K-mTOR kinase activity [16]. Further, the anti-tumor activity of BEZ235 has been shown in various cancer types originating in the liver [17], ovarian [18], colon and rectum [19,20], breast [21], and prostate [22]. Moreover, the BEZ235 has shown a better outcome in the treatment of solid tumors in the recent phase I/II clinical trials [23,24,25]. Additionally, recent reports have indicated that the combined treatment using BEZ235 with cisplatin enhanced the anti-tumor activity in hypopharyngeal squamous cell carcinoma and lung cancer [26,27]. Therefore, in this study, we set to investigate the anti-tumor effects of BEZ235 combined with regorafenib in three HCC cell lines. We explored the optimal combination of synergic anti-tumor concentrations of regorafenib and BEZ235. Our analysis showed that combined treatment with BEZ235 and regorafenib enhanced the cellular toxicity, and inhibited cell migration and invasion in HCC cells via inhibition of the Akt/mTOR pathway. 

## 2. Results

### 2.1. BEZ235 Increased the Regorafenib-Induced Inhibition of Cell Viability in HCC Cells

**Figure 1:**
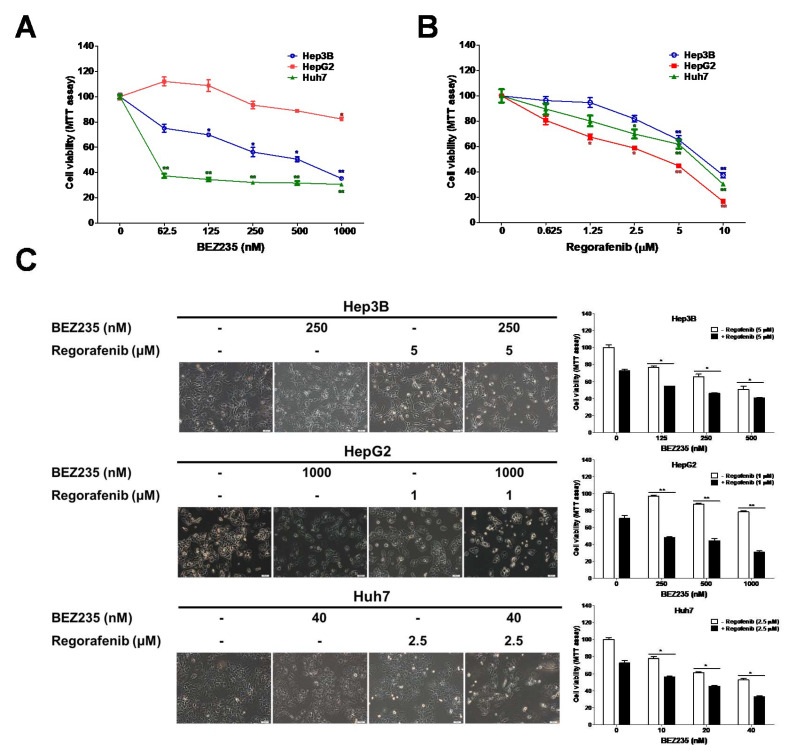
BEZ235 enhances the anti-proliferation effect of regorafenib in HCC cells. The cell viability analysis for HCC cells treated for 48 h with (**A**) BEZ235 or (**B**) regorafenib. (**C**) Cell viability analysis for the HCC cells treated with various combinations of BEZ235 and regorafenib for 48 h. Scale bar: 50 μm. Data are represented as mean ± S.D. * *p* < 0.05, and ** *p* < 0.01 versus untreated control, and BEZ235 or regorafenib alone group.

The human HCC cells, *viz.* Hep3B, HepG2, and Huh 7 were selected to establish the appropriate drug concentrations of regorafenib and BEZ235. These cells were treated with different doses of regorafenib or BEZ235 for 48 h, and their viability was analyzed using the MTT assay (Figure 1A,B). The IC_50_ values of regorafenib were found to be 7.5, 2.9, and 5.6 μM in the Hep3B, HepG2, and Huh7 cells, respectively. Additionally, we analyzed the combined effects of regorafenib and BEZ235 on the viability of HCC cells after 48 h. The combination index (CI) and dose reduction index (DRI) were calculated using the CompuSyn software. The CI < 1 indicated that the combination treatments exhibited a synergistic effect, while the DRI > 1 suggested how many folds of dose reduction can be achieved for each drug in a synergistic combination than the dose of each drug alone (Table 1). As shown in Figure 1C, the combined treatment suppressed the viability of the Hep3B (125 nM BEZ235: 54.5% ± 0.2%; 250 nM BEZ235: 46.0% ± 0.8%; 500 nM BEZ235: 40.8% ± 0.7%), HepG2 (250 nM BEZ235: 47.9% ± 1.4%; 500 nM BEZ235: 44.5% ± 2.5%; 1000 nM BEZ235: 31.1% ± 1.3%), and Huh7 (10 nM BEZ235: 56.1% ± 3.1%; 20 nM BEZ235: 45.3% ± 0.8%; 40 nM BEZ235: 32.9% ± 1.2%) cells in a dose-dependent manner than upon treatment with the BEZ235-alone. The combination of regorafenib (1 μM) and BEZ235 (1000 nM) reduced the cell viability by 68.9% in HepG2 cells (regorafenib alone: 29.4%, BEZ235 alone: 21.6%, CI: 0.45). Similar results were obtained from combination treatment with Huh7 and Hep 3B cells as well (Hep3B: regorafenib (5 μM) and BEZ235 (250 nM), CI = 0.99; Huh7: regorafenib (2.5 μM) and BEZ235 (40 nM), CI = 0.63). These doses of combination treatment were selected for further experiments in the HCC cells. 

### 2.2. BEZ235 Enhances the Regorafenib-Induced Apoptosis in HCC Cells

**Figure 2:**
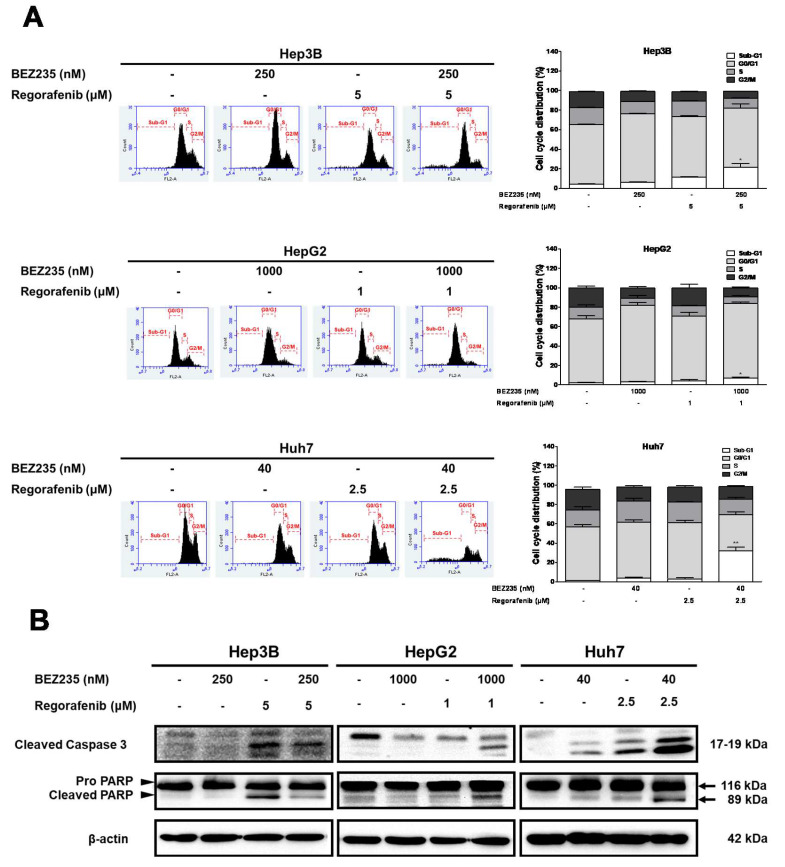
The regorafenib-induced apoptosis in HCC cells increases upon treatment with BEZ235. The flow cytometry analysis of cell cycle profiles in HCC cells treated with BEZ235 and regorafenib for 48 h (**A**). The Western blot analysis indicating the expression pattern of cleaved caspase-3 and cleaved PARP (**B**). Data are represented as mean ± S.D. * *p* < 0.05, and ** *p* < 0.01 versus untreated control, and BEZ235 or regorafenib alone group.

To test the efficacy of BEZ235 combined with regorafenib to induce apoptosis in HCC cells, cells were treated with various combination concentrations for 48 h and examined using the flow cytometry analysis and Western blot. The sub-G1 population was markedly enriched upon combined treatment of regorafenib with BEZ235 (Hep3B: 21.4% ± 3.8%, HepG2: 6.9% ± 1.3%, and Huh7: 32.0% ± 3.9%) than the control or treatment with each drug alone (Figure 2A). Further, we investigated the expression of apoptosis-associated proteins using Western blot analysis. Our results suggested that the expression of cleaved caspase-3 and cleaved PARP was increased upon the combined treatment with regorafenib and BEZ235 than that in the untreated control (Figure 2B). Taken together, these results showed that the combined treatment can significantly increase apoptosis compared to treatment with regorafenib or BEZ235 alone. Moreover, a relatively high dose of BEZ235 induced the G0/G1 growth arrest in the Hep3B and HepG2 cells than in the Huh7 cells (Figure 2A).

### 2.3. BEZ235 Increases the Regorafenib-Induced Inhibition of Cell Migration and Invasion in HCC Cells

**Figure 3:**
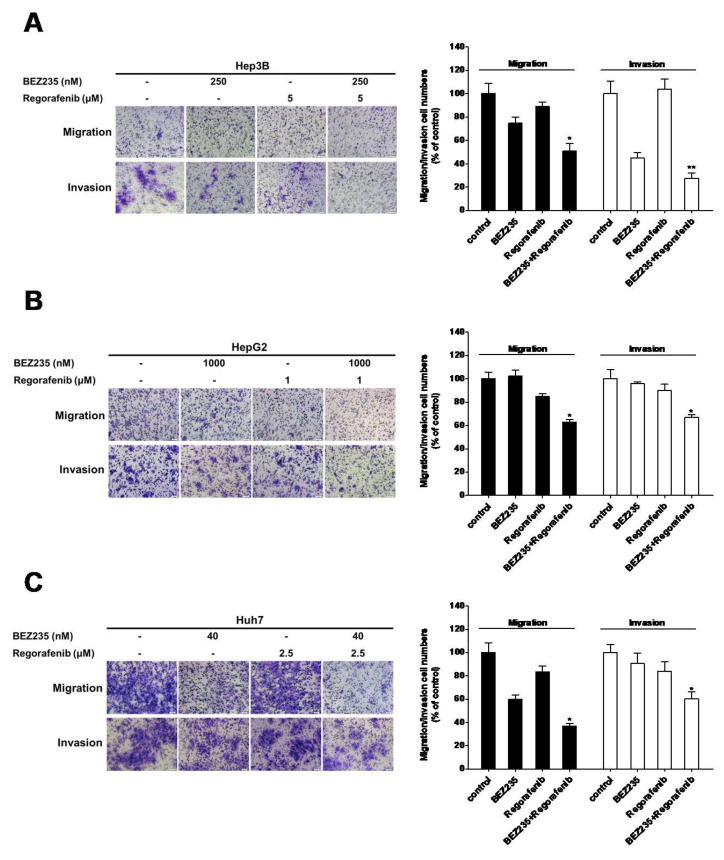
Treatment with BEZ235 enhances the anti-migration and -invasion effect of regorafenib in HCC cells. The analysis of transwell migration and invasion assay upon treatment of Hep3B (**A**), HepG2 (**B**) and Huh7 (**C**) cells with BEZ235 and regorafenib for 24 h or 48 h. Data are represented as means ± S.D. * *p* < 0.05, and ** *p* < 0.01 versus untreated control, BEZ235 or regorafenib alone group.

The cancer cells exhibit cell migration and invasion that contribute to their metastatic behavior. Therefore, the transwell assays were performed to assess the inhibitory effects of BEZ235 combined with regorafenib on the cell motility of HCC cells. The HCC cells were treated, with or without BEZ235 or regorafenib, for 24 h or 48 h followed by the transwell assay for analysis of migration or invasion, respectively (Figure 3). The combined treatment of BEZ235 and regorafenib suppressed the migration of HCC cells (Hep3B: 50.9% ± 6.3%, HepG2: 62.9% ± 2.2%, and Huh7: 36.9% ± 2.3%) more than the drug alone or in the control group. Moreover, the combined treatment suppressed the invasion in HCC cells (Hep3B: 27.7% ± 4.4%, HepG2: 67.2% ± 1.9%, and Huh7: 60.5% ± 5.7%) more than the drug alone or in the control group. Taken together, these results showed that the combined treatment significantly inhibited the migration and invasion ability of HCC cells.

### 2.4. Combined Drug Treatment Affects the Expression of EMT-Associated Proteins in HCC Cells

**Figure 4:**
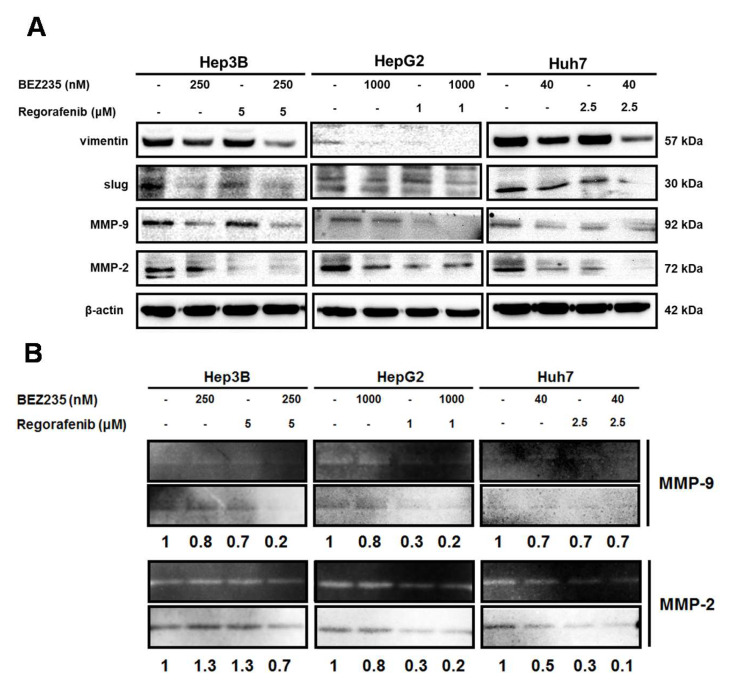
EMT-associated proteins are down-regulated upon combinatorial treatment in HCC cells. The expression pattern of the EMT-associated proteins was analyzed using Western blot (**A**), while the enzymatic activity of MMP-9/-2 were checked using zymography (**B**), following combined treatment of the HCC cells.

Next, we analyzed the expression of the EMT-associated proteins to understand the mechanism of regulation of cell migration and invasion upon the combined treatment. The Western blot analysis indicated down-regulation of the expression of vimentin, MMP-9, MMP-2 and slug in HCC cells after the combined drug treatment for 48 h (Figure 4A). Additionally, the combined treatment reduced the enzymatic activity of MMP-9 and MMP-2 in HCC cells in the zymography analysis (Figure 4B). Taken together, these results suggested that the combined treatment inhibited the EMT to suppress cell migration and invasion of HCC cells.

### 2.5. Regorafenib and BEZ235 Suppress the Akt/mTOR Pathway in the HCC Cells

**Figure 5:**
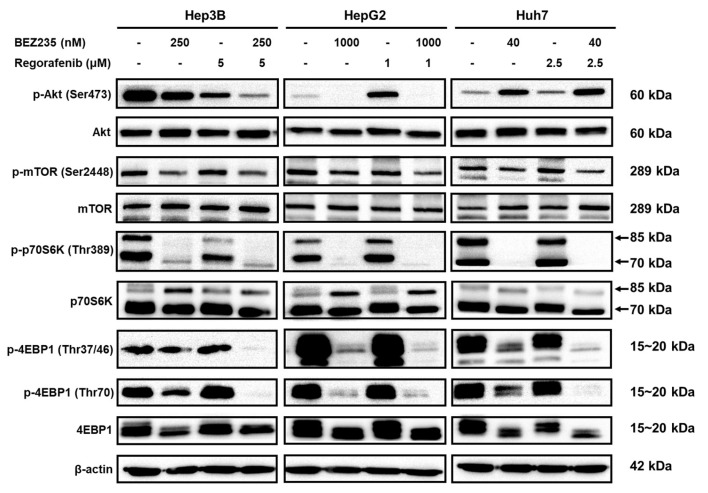
The combined treatment inhibits the activation of Akt/mTOR signaling pathway in the HCC cells. The panels indicate Western blot analysis for phosphorylation and total protein levels of the members of the Akt/mTOR pathway in the HCC cells treated with BEZ235 and regorafenib for 48 h.

Further, to investigate whether the anti-tumor effects of the combined treatment are mediated via the inhibition of the Akt/mTOR pathway, the phosphorylation pattern of the members of this pathway were analyzed in the HCC cells treated with BEZ235 and regorafenib for 48 h. As shown in Figure 5, the phosphorylation of Akt (Ser473) was inhibited in the Hep3B cells, while induced in the HepG2 cells, upon treatment with regorafenib-alone. However, combination treatment with BEZ235 suppressed the phosphorylation of Akt in both the cells. Furthermore, the phosphorylation status of the mTOR, p70S6K and 4EBP1 in the HCC cells was not affected upon treatment with regorafenib-alone more than in the control group. Additionally, treatment of the HCC cells with BEZ235-alone or combined with regorafenib significantly reduced the levels of p-mTOR (Ser2448), p-p70S6K (Thr389), p-4EBP1 (Thr37/46) and p-4EBP1 (Thr70), in contrast with the regorafenib-alone or control group. Thus, these results indicated that the combined treatment regulated the anti-tumor activity by suppressing the Akt/mTOR signaling pathway in the HCC cells.

## 3. Discussion 

The regorafenib is the only second-line treatment available for the treatment of the patients intolerant to sorafenib. However, given its high cost and adverse effects in the patients, it is restricted in patients with mild or moderate hepatic impairment (Child-Pugh A and B) and sorafenib-intolerance. Additionally, the HCC is highly therapy-resistant, and hence requires systemic therapies against the multiple oncogenic pathways [28,29]. Therefore, it is imperative to develop new combined therapy using regorafenib and other alternative drugs. A previous study observed that the combination of BEZ235 with cytotoxic agents, such as doxorubicin, cisplatin, 5-FU or irinotecan were more effective in increasing the cytotoxicity in the HCC [30]. In this study, the MTT assay analysis indicated that a low concentration of the BEZ235 (40 nM–1 μM) enhanced the regorafenib-induced growth inhibition in the HCC cells. The CI values in all the HCC cells were below the line of additivity (CI < 1), indicating the synergistic effect of BEZ235 and regorafenib. Moreover, all the DRI values obtained from the combination treatments were positive, suggesting that the reduction in the concentration of both the drugs was allowed and the reduction in their toxicity could be expected. The combined treatment induced the enrichment of the sub-G1 cell population and enhanced the pro-apoptotic effect along with an increase in the expression of cleaved caspase-3 and cleaved PARP in HCC cells. Furthermore, the G0/G1 cell cycle arrest induced by regorafenib treatment was elevated upon the combined treatment with BEZ235. The combination treatments showed a slight growth inhibition effect on RWPE-1 (human prostate epithelial) cells (data not shown). In addition, a recent study reported that the IC_50_ values of BEZ235 for normal human fibroblast and normal human bronchial epithelial cells were greater than 10 μM [31]. Thus, there might be some discrepancy effect of combination treatments in different non-tumoral cell lines that is worth further clarification.

The MMPs, vimentin and slug participate in the EMT to promote cell motility, via cell adhesion, migration and invasion [32,33]. The disruption of the basement membranes by the MMPs, such as MMP-9/-2, lead to the tumor cell migration and invasion, and thus play crucial roles in the EMT processes [34,35]. Further, the treatment with BEZ235 down-regulated the expression of MMP-9/-2 to suppress the cancer cell migration and invasion [36,37]. Moreover, treatment with regorafenib diminished the expression of the MMP-9/-2 and inhibited cell invasion in the HCC SK-Hep1 cells [38]. Here, our analysis indicated that the combined treatment with BEZ235 and regorafenib inhibited cell migration and invasion better than treatment with either agent alone. Furthermore, the tumor cell invasion is facilitated by the activation of the Src/slug signaling that induces the expression of MMP-9/-2 and vimentin during the EMT [39,40]. Our analysis suggested that the expression of MMP-9/-2, vimentin and slug was down-regulated in the HCC cells in response to the combined treatment. Thus, the combined treatment with BEZ235 and regorafenib attenuated the EMT in HCC cells via inhibition of the slug/MMP-9/MMP-2 and vimentin signaling axis. 

Since the PI3K/Akt/mTOR signaling pathway is often dysregulated in several human malignancies, inhibitor targeting of this pathway holds immense interest in cancer treatment [41]. Further, recent studies have indicated that the blockade of the mTOR signaling pathway inhibited cell viability and motility of HCC cells [42,43]. Additionally, regorafenib has been shown to act as a weak inhibitor of the PI3K/Akt pathway, and hence their activation may cause resistance to regorafenib [44]. Thus, the combined treatment that has the ability to simultaneously inhibit multiple signaling pathways, including the PI3K/Akt/mTOR pathway, would be required. For instance, the combined treatment using regorafenib and chemical compounds, such as the chlorogenic acid (CGA) and cisplatin, enhanced its cytotoxicity by suppressing the PI3K/Akt/mTOR pathway in the HCC cells [45,46]. Furthermore, treatment with BEZ235 was found to suppress the cancer cell proliferation, migration, and invasion by regulating the Akt/mTOR pathway [19,47]. Moreover, the combined treatment with BEZ235 and chemotherapy drugs, such as cisplatin, trametinib and paclitaxel, provided a synergistic effect by suppressing the Akt/mTOR pathway in cancer [26,48,49]. Hence, the combined treatment with BEZ235 and regorafenib could significantly suppress the activation of p-mTOR (ser2448), p-p70S6K (Thr389), p-4EBP1 (Thr37/46) and p-4EBP1 (Thr70) in the HCC cells. The treatment with regorafenib-alone enhanced Akt phosphorylation in the HepG2 cells, which was dramatically suppressed by the combined treatment with BEZ235. Conversely, treatment with BEZ235 enhanced the phosphorylation of Akt in Huh7 cells, consistent with the report by Ou and colleagues [50]. However, BEZ235 effectively suppressed the phosphorylation of downstream effectors, such as the mTOR, p70S6K and 4EBP1. 

Serra and colleagues reported that BEZ235 (40 mg/kg/day) showed great anti-tumor activity both in cellular models and in breast cancer xenografts [51]. A recent study also indicated that BEZ235 (45 mg/kg/day) displayed promising therapeutic efficiency against paclitaxel-resistant gastric cancer in vivo [52]. In addition, Roulin and colleagues reported that the simultaneous use of BEZ235 (30 mg/kg/day) with sorafenib has greater antitumor benefits compared to either drug alone in renal cell carcinoma xenografts [53]. However, the safety, efficiency and mechanism of action of this combination treatment need to be elucidated in further investigation by in vivo experiments.

**Figure 6:**
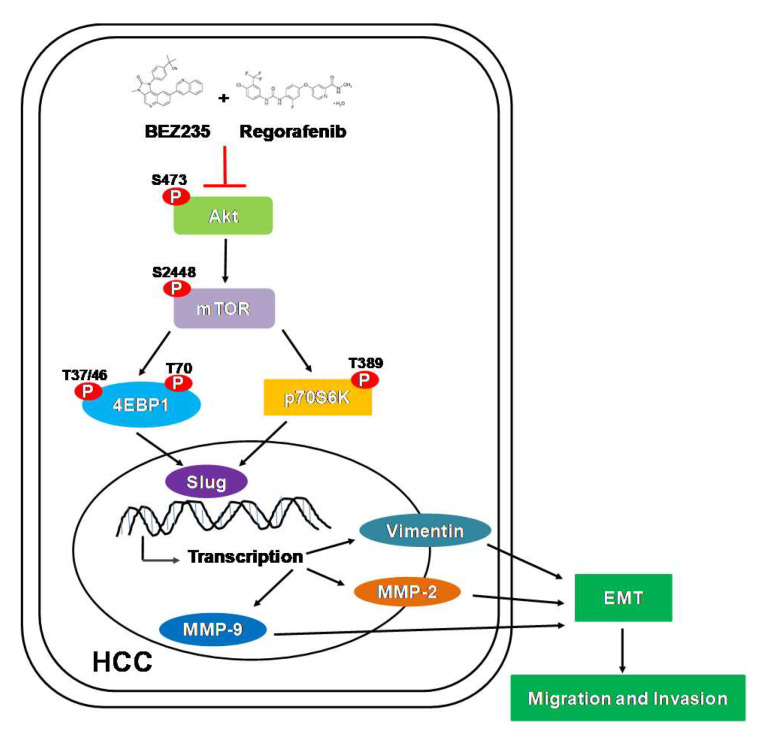
Model depicting the anti-tumor effect of the combined treatment with BEZ235 and regorafenib in HCC cells. The combined treatment suppressed HCC cell migration and invasion through inhibition of the Akt/mTOR signaling pathway.

In conclusion, our study described the anti-tumor effect of BEZ235 in combination with regorafenib in the human HCC cells: Hep3B, HepG2 and Huh7. The combined treatment enhanced the anti-proliferation and pro-apoptotic activity of the individual drugs. Additionally, the transwell assay indicated better inhibition of the cell motility upon combined treatment. Moreover, the expression of the EMT-related proteins was reduced and the MMP-9/-2 enzymatic activity was diminished following the combined treatment. Furthermore, treatment with regorafenib and BEZ235 significantly inhibited the phosphorylation of Akt, mTOR and p70S6K proteins. Taken together, as summarized in Figure 6, our study suggests that a combined treatment with BEZ235 and regorafenib may prove to be an effective strategy for the treatment of HCC.

## 4. Materials and Methods

### 4.1. Chemicals and Antibodies

BEZ235 was purchased from LC laboratories (Woburn, MA, USA) and regorafenib was obtained from Toronto Research Chemicals (North York, ON, Canada). Other chemicals were purchased from Sigma Chemical Co. (St. Louis, MO, USA). The antibodies were all purchased from Cell Signaling Technology, Inc. (Danvers, MA, USA). Bradford protein assay kit was purchased from Bio-Rad (Hercules, CA, USA). PVDF membranes were purchased from Merck Millipore (Bedford, MA, USA). The Western blot chemiluminescence reagents were purchased from Amersham Biosciences (Arlington Heights, IL, USA).

### 4.2. Cell Culture

The HCC cell lines Hep3B, HepG2 and Huh7 were purchased from BCRC (Bioresource Collection and Research Center, Hsinchu, Taiwan). All cell lines were authenticated annually using STR analysis and were tested negative for mycoplasma. Cells were cultured in an appropriate medium according to the suggestions from the BCRC website. All media components were purchased from Invitrogen (Carlsbad, CA, USA). 

### 4.3. MTT Assay

The cell viability was evaluated using MTT assay, as previously described [54,55]. 

### 4.4. Western Blot Analysis 

Western blot analysis was performed as previously described [54,55]. 

### 4.5. Cell Migration and Invasion Assay

Cell migration and invasion assays were performed as previously described [55]. Briefly, cells (5 × 10^4^) were treated with indicated drugs for 24 h then were plated in the upper chambers (8 μm pore size hanging inserts) in a 24-well plate for 24 h. Meanwhile, cultured medium containing 10% FBS was used as a chemo-attractant in the lower chambers. The non-migrated cells on the upper surface of the chamber were removed and the invaded cells were fixed with 10 % formalin and stained with 0.2% crystal violet for 15 min. The invaded cells were counted and photographed at 200 × magnification.

Invasion assay: Cells were seeded onto Matrigel-coated (2 mg/mL) upper chambers and followed by migration assay protocol. 

### 4.6. Flow Cytometric Analysis

The cell cycle was determined by flow cytometry analysis as previously described [54]. Briefly, approximately 3 × 10^5^ cells were incubated with or without BEZ235 in the presence or absence of regorafenib for 48 h. Cells were stained with 20 μg/mL propidium iodide solution for cell cycle analysis, and apoptotic cell death was examined using annexin V-FITC detection kits according to the manufacturer’s instructions (BD Biosciences, San Diego, CA, USA). The data were collected and analyzed by the BD Accuri C6 flow cytometer.

### 4.7. Gelatin Zymography

Gelatin zymography was performed as previously described [55]. Briefly, cells were seeded in 6-well plates and treated with indicated drugs for 48 h and the supernatant was collected. The supernatant was analyzed using electrophoresis—gels were stained and gelatinolytic activity was shown as clear areas. 

### 4.8. Statistical Analysis

All data were shown as mean ± S.D. Statistical differences were analyzed using the Student’s *t*-test for normally distributed values.

## Figures and Tables

**Table 1:** CI and DRI of BEZ235 and Regorafenib combination in HCC.

**Concentration**		**Hep3B**		**DRI**	
**BEZ235 (nM)**	**Regorafenib (μM)**	**fa**	**CI**	**BEZ235**	**Regorafenib**
125	5	0.46	1.04	3.46	1.34
250	5	0.54	0.99	2.53	1.68
500	5	0.59	1.13	1.61	1.94
**Concentration**		**HepG2**		**DRI**	
**BEZ235 (nM)**	**Regorafenib (μM)**	**fa**	**CI**	**BEZ235**	**Regorafenib**
250	1	0.52	0.43	8.59	3.16
500	1	0.56	0.48	4.75	3.70
1000	1	0.69	0.45	3.38	6.42
**Concentration**		**Huh7**		**DRI**	
**BEZ235 (nM)**	**Regorafenib (μM)**	**fa**	**CI**	**BEZ235**	**Regorafenib**
10	2.5	0.44	0.88	3.13	1.77
20	2.5	0.55	0.74	2.67	2.74
40	2.5	0.67	0.63	2.47	4.53

Abbreviation: CI, combination index; DRI, dose reduction index; fa, fraction affected.
